# Correlations between Sagittal Parameters and Functional Scores in 65-Year-Old Osteoporotic Females with Vertebral Body Fracture under Low-Energy Mechanism

**DOI:** 10.3390/jcm13030774

**Published:** 2024-01-29

**Authors:** Szu-Wei Chen, Wen-Tien Wu, Ru-Ping Lee, Tzai-Chiu Yu, Ing-Ho Chen, Jen-Hung Wang, Kuang-Ting Yeh

**Affiliations:** 1 School of Medicine, Tzu Chi University, Hualien 970374, Taiwan; 108311145@gms.tcu.edu.tw (S.-W.C.); timwu@tzuchi.com.tw (W.-T.W.); feyu@tzuchi.com.tw (T.-C.Y.); ihchen@tzuchi.com.tw (I.-H.C.); 2 Department of Orthopedics, Hualien Tzu Chi Hospital, Buddhist Tzu Chi Medical Foundation, Hualien 970473, Taiwan; 3 Institute of Medical Sciences, Tzu Chi University, Hualien 970374, Taiwan; fish@gms.tcu.edu.tw; 4 Department of Medical Research, Hualien Tzu Chi Hospital, Buddhist Tzu Chi Medical Foundation, Hualien 970473, Taiwan; paulwang@tzuchi.com.tw; 5 Department of Medical Education, Hualien Tzu Chi Hospital, Buddhist Tzu Chi Medical Foundation, Hualien 970473, Taiwan; 6 Graduate Institute of Clinical Pharmacy, Tzu Chi University, Hualien 970374, Taiwan

**Keywords:** osteoporotic fractures, spinal fractures, vertebral compression fractures, thoracolumbar cobb angle, sagittal alignment, receiver operating characteristic (ROC) curve method, Oswestry disability index, visual analogue scale, older adults

## Abstract

**Background**: Vertebral compression fractures (VCFs) are the most common fragility fractures associated with low-energy injury mechanisms in postmenopausal women with osteoporosis. No clear consensus is currently available on the optimal timing for surgical intervention in specific cases. **Methods**: This study examined the correlations between sagittal parameters, functional scores, and the appropriate timing for surgical intervention during the recovery stage in patients with osteoporosis with thoracolumbar (TL) vertebral body fractures. A total of 161 women aged ≥ 65 years with osteoporosis were included in the study. Spinal sagittal parameters from standing plain films and functional outcomes as the Oswestry disability index (ODI) and the visual analogue scale (VAS) were collected. **Results**: We found that TL junction Cobb angle was significantly correlated with ODI > 30 (*p* < 0.001) and VAS > 6 (*p* < 0.001) and the discriminative values for predicting ODI > 30 and VAS > 6 were a TL kyphotic angle of 14.5° and 13.5°, respectively. Among women aged ≥ 65 years with osteoporosis, the back pain and functional impairment observed within 6 months following a compression fracture are associated with a greater TL kyphosis angle. **Conclusions**: This suggests that a more proactive approach may be necessary when addressing the conditions of these patients.

## 1. Introduction

In the elderly, the incidence of fragility fractures is rising, with studies showing a strong link between osteoporosis and chronic diseases like diabetes, hypertension, heart disease, and thyroid disorders, particularly hyperthyroidism and treated hypothyroidism [1]. Research reveals that thyroid diseases, especially when treated with hormone replacement therapy such as levothyroxine, contribute to metabolic changes affecting bone mineral density and increasing fragility fracture risk [2]. Vertebral compression fractures (VCFs) are the most common fragility fractures associated with low-energy injury mechanisms in postmenopausal women with osteoporosis [3]. Following the occurrence of VCFs, patients experience back pain, limitations in the performance of daily activities, an increased risk of recurrent fractures, the development of back deformities, and even increased mortality rates [3,4,5,6,7]. Recent studies have highlighted the pivotal role of sagittal balance in the etiology of VCFs, revealing that elderly individuals with thoracic kyphosis and a reduction in lumbar lordosis are more prone to VCFs, regardless of the presence of osteoporosis [8]. Conversely, the occurrence of VCFs induces changes in sagittal parameters. Research has indicated that even a single VCF can alter spinal and pelvic parameters as well as the sagittal balance of the spine [8]. Moreover, the number and severity of VCFs are significantly correlated with global sagittal alignment [9]. In VCF cases, vertebral body compression or burst can lead to kyphosis over time, accompanied by expected compensatory mechanisms, including increased lumbar spine lordosis, posterior tilting or rotation of the pelvis, hip extension, and even knee flexion and dorsiflexion of the ankles [9]. Both VCFs and sagittal imbalance contribute to the decreased performance of activities of daily living (ADLs) and diminished quality of life (QOL) [10,11]. As society ages, osteoporosis and the potential occurrence of VCFs have become critical social concerns.

Therefore, examining the relationship between sagittal alignment and functional performance and the importance of early intervention following VCF occurrence is crucial. Research has focused on the impact of VCFs on sagittal balance, and research on various factors and conditions, including spinal sagittal parameters, is limited. This gap underscores the necessity for more proactive approaches in the management of VCFs following their onset. Therefore, this study examined the correlations between sagittal parameters and functional scores in patients with osteoporosis with vertebral body fractures during the recovery stage.

## 2. Materials and Methods

### 2.1. Study Design

This observational prospective single-center study was approved by the Ethics Committee of the Institutional Review Board of Hualien Tzu Chi Hospital, Buddhist Tzu Chi Medical Foundation. A total of 161 women aged > 65 years were included in the study, all of whom met the criteria for osteoporosis (as defined by a dual-energy X-ray absorptiometry (DEXA) scan T score of less than −2.5) and had experienced a new-onset compression fracture over a single level of the vertebral body in the thoracolumbar spine caused by a low-trauma mechanism. The patients had no history of spine fusion or major lower limb joint surgery and could stand up and walk for at least 50 min. Notably, prior to the fractures, none of the patients reported experiencing any back pain. Following the fracture, all the patients were prescribed pain medication and were advised to wear a back brace.

Demographic information of the participants was collected, including age and body mass index (BMI). Standing whole spine triple films were captured 6–12 months posttrauma to obtain spinal sagittal parameters. Functional outcomes were assessed using the Oswestry disability index (ODI) and visual analogue scale (VAS) during the posttrauma 6–12-month period. Each patient’s bone mineral density was measured using a DEXA scan according to the World Health Organization, with a T score of <2.5 SD indicative of osteoporosis. The standing whole spine triple film was arranged 6 months after the acute VCF onset to obtain spinal sagittal parameters from the plain film.

### 2.2. Spinal Sagittal Parameters

**Figure 1:**
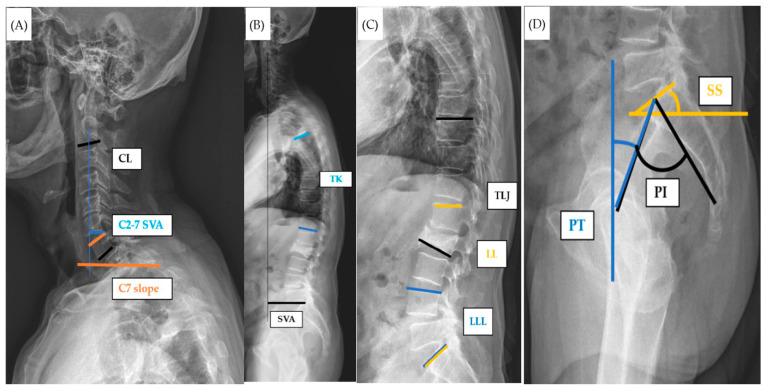
The measurement methods of the sagittal alignment parameters. (**A**) CL: cervical lordosis; C2–7 SVA: sagittal vertical axis; C7 slope (**B**) TK: thoracic kyphosis; SVA: sagittal vertical axis. (**C**) LL: lumbar lordosis; LLL: lower lumbar lordosis; TLJ: thoracolumbar junction kyphotic angle. (**D**) PI: pelvic incidence; SS: sacral slope; PT: pelvic tilt.

The sagittal parameters were introduced and defined as follows [12,13] (Figure 1A–D): cervical lordosis (CL) is the angle, measured in degrees, formed between the lower endplate of the second cervical vertebra (C2) and the lower endplate of C7. C7 slope is determined by measuring the angle between a horizontal reference line and a line that runs parallel to the upper endplate of C7. C2–7 sagittal vertical axis (SVA) is determined by measuring the horizontal distance between the posterosuperior corner of the C7 vertebral body and a vertical line drawn from the centroid of C2 (Figure 1A). SVA is the length of a horizontal line connecting the posterior superior sacral end plate to a vertical plumbline dropped from the centroid of the C7 vertebral body. Thoracic kyphosis is the angle, measured in degrees, formed between the upper endplate of the fifth thoracic vertebra (T5) and the lower endplate of T12 (Figure 1B). Lumbar lordosis (LL) refers to the angle, measured in degrees, formed between the upper endplate of the first lumbar vertebra (L1) and the upper endplate of the first sacral vertebra (S1). Lower lumbar lordosis (LLL) is the angle, measured in degrees, formed between the upper endplate of the fourth lumbar vertebra (L4) and the upper endplate of the S1. Thoracolumbar junction kyphotic angle (TL junction) refers to the angle, measured in degrees, formed between the upper endplate of the tenth thoracic vertebra (T10) and the lower endplate of the second lumbar vertebra (L2) (Figure 1C). Pelvic incidence (PI) is the angle between the line perpendicular to the sacral endplate at its midpoint and a line connecting this point to the axis of the femoral head. The sacral slope (SS) is the horizontal and sacral plate angle. Pelvic tilt (PT) is the angle between a vertical reference line and a line from the midpoint of the sacral endplate to the femoral rotational axis (Figure 1D). PI and LL mismatch (PI-LL) is the value obtained by subtracting lumbar lordosis from pelvic incidence.

### 2.3. Statistical Analysis

All statistical analyses were conducted using SPSS v23.0 (IBM, New York, NY, USA). Demographic information is expressed as mean ± standard deviation, along with minimum and maximum values. Multiple logistic regression analysis was employed to determine the correlation between risk factors and unfavorable functional outcomes, specifically defined as ODI > 30 [14] or VAS > 6 [15]. The results are presented as odds ratios (ORs) with corresponding 95% confidence intervals (CIs). A *p* value ≤ 0.05 was considered statistically significant after testing. Additionally, we employed a receiver operating characteristic (ROC) curve analysis to identify the optimal threshold for spinal sagittal parameters. Sensitivity, specificity, and area under the curve were also calculated.

## 3. Results

There were 161 female patients included in this study. Of them, 62 had VCF over T10-L2, 51 of them had VCF over L3–5, and the other 48 of them had VCF over T5–9. The average age was 72.71 ± 5.61 years, and the average BMI was 26.48 ± 5.14 kg/m^2^ (Table 1). The specific sagittal parameters and functional outcome measures are as follows: CL: 18.61 ± 14.23°; C7 slope: 28.52 ± 11.1°; C2–7 SVA: 17.21 ± 11.53 mm; LL: 38.99 ± 18.4°; LLL: 30.18 ± 13.15°; TL junction: 15.47 ± 10.68°; SVA: 64.75 ± 41.95 mm; PI: 55.13 ± 14.14°; SS: 32.76 ± 12.52°; PT: 22.37 ± 9.98°; PI-LL: 18.48 ± 13.79. Regarding functional outcome, the ODI was 24.8 ± 9.38, and the VAS score was 5.25 ± 2.29 (Table 1).

We defined ODI > 30 and VAS > 6 as indicators of poor functional outcomes and conducted a multiple logistic regression analysis to identify the factors associated with these outcomes. The results revealed a significant correlation between the TL junction and both ODI > 30 (OR = 1.28, 95% CI: 1.18–1.39, *p* < 0.001; Table 2) and VAS > 6 (OR = 1.17, 95% CI: 1.10–1.23, *p* < 0.001; Table 3), indicating that a higher thoracolumbar kyphotic angle is associated with an increased risk of poor functional outcomes. Among the 161 patients, 72 were aged > 72 years, and 68 were classified as being obese. In the stratified analysis based on patient age and BMI, the TL junction remained significantly correlated with ODI > 30 (all *p* < 0.001; Table 4) and VAS > 6 (all *p* < 0.001; Table 5) across all the stratified groups. Additionally, LL was inversely correlated with ODI > 30 in patients younger than 72 years (OR = 0.89, 95% CI: 0.80–0.99, *p* = 0.043), and PT was inversely correlated with ODI > 30 in nonobese patients (OR = 0.92, 95% CI: 0.85–0.99, *p* = 0.036; Table 4). Furthermore, CL was inversely correlated with VAS > 6 in patients younger than 72 years (OR = 0.85, 95% CI: 0.76–0.96, *p* = 0.007), whereas C2–7 SVA was inversely correlated with VAS > 6 in the nonobese patients (OR = 0.92, 95% CI: 0.85–0.99, *p* = 0.036; Table 5).

**Figure 2:**
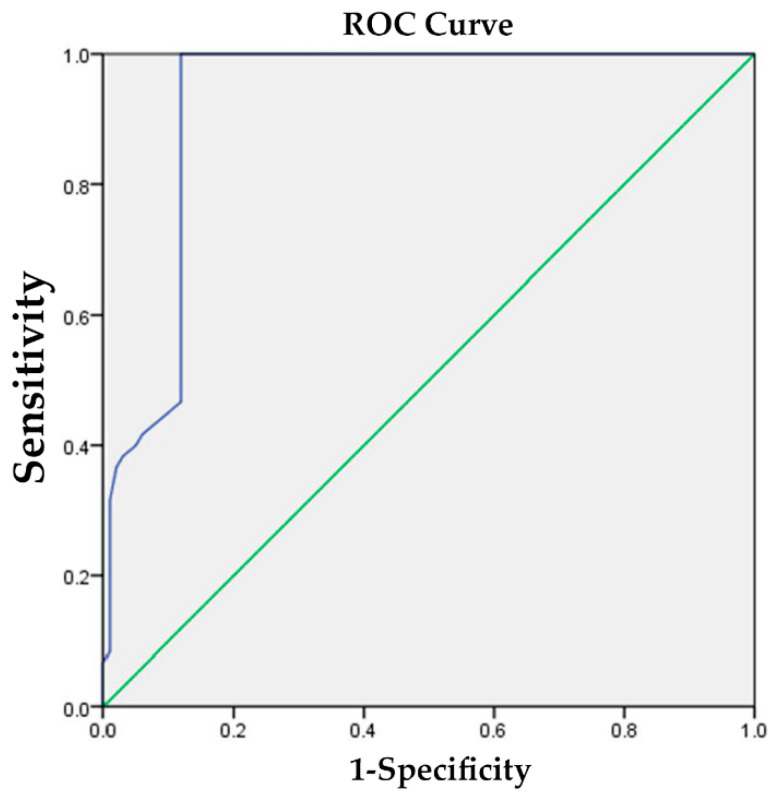
The discriminative value for identifying poor functional outcomes, as assessed by the area under curve (AUC) method, was 0.927 for ODI; green line: reference line; blue line: Oswestry disability index (ODI).

**Figure 3:**
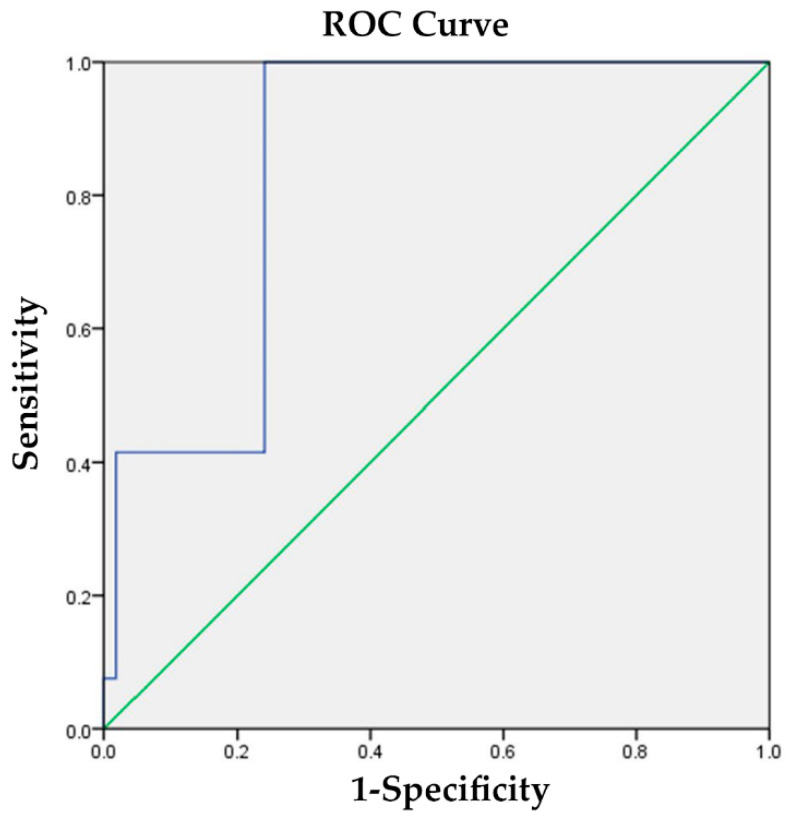
The discriminative value for identifying poor functional outcomes, as assessed by the area under curve (AUC) method, was 0.853 for VAS; green line: reference line; blue line: visual analogue scale (VAS) for back pain.

The TL junction kyphotic angle exhibited variations between patients with and without poor functional outcomes. The discriminative values, as determined using the area under the curve, were 0.927 for ODI > 30 and 0.853 for VAS > 6 (Figure 2 and Figure 3). For ODI > 30, a thoracolumbar kyphotic angle of 14.5° yielded a sensitivity of 1.000 and a specificity of 0.881. Similarly, for VAS > 6, a thoracolumbar kyphotic angle of 13.5° yielded a sensitivity of 1.000 and a specificity of 0.759.

## 4. Discussion

This study investigated the association between sagittal parameters and functional performance in postmenopausal women aged ≥ 65 years with osteoporosis during the recovery phase following a VCF. Although most VCFs typically heal within 2–3 months, more severe fractures, the presence of osteoporosis, and patient noncompliance with medical instructions lead to a longer healing process. During the recovery period, some patients are bedridden, and it can take up to 6 months after the fracture before the patients are able to stand. Due to this timeline, we obtained standing whole spine triple films 6–12 months after the fracture.

This study revealed a significant association between a greater thoracolumbar kyphotic angle and elevated scores on both the ODI (OR = 1.28, 95% CI: 1.18–1.39, *p* < 0.001) and VAS (OR = 1.17, 95% CI: 1.10–1.23, *p* < 0.001). Notably, when stratifying the participants under different conditions, such as age (≤72 versus >72 years) and obesity status (nonobese versus obese), the significance of these findings persisted within all subgroups. However, previous research has suggested an association between being underweight in patients with VCF and a reduced ability to perform ADLs, indicating a poorer prognosis [16]. These findings suggest that individuals with a greater thoracolumbar kyphotic angle tend to exhibit less satisfactory functional performance and experience more pain following a VCF.

The study’s findings are consistent with previous research indicating that sagittal imbalance and postural deformities resulting from VCFs can also impair ADLs and reduce the overall quality of life [10,17,18,19]. In response to increased kyphosis, individuals may adopt various postural compensations, including lumbar spine lordosis, posterior tilting or rotation of the pelvis, hip extension, and even knee flexion and dorsiflexion of the ankles. Maintaining such postural compensations demands higher energy expenditure than maintaining a normal posture. Over the long term, this may contribute to chronic back pain, an elevated risk of falls, or secondary osteoarthritis of the knees [20].

The treatment objectives for individuals with VCFs include alleviating or eliminating pain, restoring functionality, and preventing further fractures. Typically, stable injuries are initially addressed through conservative measures, which may include pain medications, temporary reduction in activity, the use of a back brace, and medications aimed at preventing additional fractures [21]. A previous study revealed the promising potential of integrating shockwave therapy into LBP rehabilitation protocols, considering its anti-inflammatory, pain-relieving, muscle-relaxing, and nerve-fiber-remodeling effects [22]. Currently, no conclusive evidence supporting the superiority of any specific conservative management protocol is available [23], and the methods recommended by various guidelines are inconsistent [24]. Moreover, some conservative treatments lack robust evidence for support, and their efficacy remains unknown [25,26]. One study identified the incorrect use of a brace as a risk factor for VCF progression [27].

Surgical options for treating VCFs include vertebral augmentation procedures, such as vertebroplasty and kyphoplasty [21,28]. In individuals with VCFs, vertebral augmentation procedures have been shown to enhance spinal function and alleviate pain, with comparable mortality and morbidity rates to nonsurgical management [29,30]. These interventions may be considered for individuals with painful VCFs characterized by significant kyphotic deformity or disability, particularly when patients do not respond to initial conservative management within a 4–6-week timeframe [21,31]. Studies have also suggested that surgery is a viable option for patients who were initially treated conservatively but are experiencing a progressive increase in kyphosis (>10° compared with discharge X-ray) [23]. However, no clear consensus is currently available on the timing of surgical intervention in the management of VCFs.

The inverse correlation between LL and ODI > 30 in patients younger than 72 years suggests that in this demographic, a decrease in LL is associated with a higher disability score. This finding is consistent with the literature, which indicates that a reduced LL can lead to abnormal spinal alignment, increasing strain on the lumbar vertebrae and exacerbating pain and disability. For example, Imagama et al. demonstrated the importance of LL in maintaining sagittal balance and its impact on the quality of life in elderly populations [10]. Furthermore, a study by Cho et al. highlighted that alterations in LL can significantly affect postural stability and contribute to increased disability [32]. The inverse correlation observed between PT and ODI > 30 in nonobese patients suggests that higher PT is associated with lower disability scores in this group. This could be interpreted as a compensatory mechanism wherein increased PT helps maintain sagittal balance in the absence of obesity-related factors that might otherwise exacerbate spinal misalignment. Lafage et al. observed similar patterns, indicating that PT is a crucial parameter in spinal balance, particularly in nonobese individuals where adipose tissue does not significantly influence spinal curvature [33]. The inverse correlation observed between CL and VAS > 6 in patients younger than 72 years aligns with studies suggesting that reduced cervical lordosis can contribute to increased cervical pain and overall discomfort. Research by Li et al. emphasizes the critical role of cervical sagittal alignment in neck pain and overall spinal health, underscoring the importance of maintaining cervical curvature to manage pain levels in younger patients with spinal pathologies [34]. Similarly, the inverse correlation between C2–7 SVA and VAS > 6 in nonobese patients adds to the growing body of evidence supporting the role of sagittal balance in spinal health. A forward shift in the sagittal vertical axis, as indicated by an increase in C2–7 SVA, has been linked to worse clinical outcomes in terms of pain and disability. Perez et al. demonstrated the importance of considering BMI when optimizing sagittal alignment in patients undergoing cervical fusion [35], and these correlations may be more pronounced in the nonobese population, aligning with our findings.

To determine the circumstances under which surgical intervention should be considered, we employed ROC curve analysis to identify the optimal cutoff value for poor functional outcomes. For a poor ODI (ODI > 30), a thoracolumbar kyphotic angle of 14.5° demonstrated a sensitivity of 1.000 and a specificity of 0.881. Similarly, for a severe VAS (VAS > 6), a thoracolumbar kyphotic angle of 13.5° exhibited a sensitivity of 1.000 and a specificity of 0.759. Therefore, we established 14° (the average value of 14.5° and 13.5°) as the best cutoff point for defining poor functional outcomes. This threshold can be considered the opportune moment for surgical intervention. The presence of sagittal imbalance at 6 months, associated with higher ODI and VAS scores, suggests that initiating early active treatment is advisable when the thoracolumbar kyphotic angle, acquired in either a standing or lying position, exceeds the critical threshold of 14°. It is expected that the thoracolumbar angle when standing will be greater than that when lying down. In such cases, a transition from conservative treatment to surgical intervention, such as vertebral augmentation or PI correction, may be warranted. Previous research has demonstrated that, under specific conditions, patients who undergo surgery tend to experience superior pain reduction and an improved quality of life than those treated with nonsurgical management [36,37,38]. However, future research should explore the outcomes following surgery in patients with a greater thoracolumbar angle.

This study has some limitations that should be acknowledged. First, we did not account for the potential variations in the effects of VCFs occurring at different locations in the spine on patients. VCFs most commonly occur near the thoracolumbar junction, a transition zone that is less flexible than other spinal segments. Consequently, our study results indicated that the thoracolumbar kyphotic angle had the most substantial impact on functional scores among all sagittal parameters. Second, although we excluded patients with significant lower limb surgery from the analysis, we did not consider compensatory mechanisms such as knee flexion and dorsiflexion of the ankle in subsequent assessments. These mechanisms could also be factors influencing the functional outcomes of patients. Despite these limitations, the study’s results underscore the significant impact of thoracolumbar kyphotic change caused by VCFs and provide an optimal cutoff value for poor functional outcomes. These findings offer a valuable reference for treatment decisions during the initial and recovery stages of VCFs in older female patients.

## 5. Conclusions

In postmenopausal women aged ≥ 65 years with osteoporosis who experience vertebral body fractures, a greater thoracolumbar kyphotic angle was associated with higher ODI and VAS scores. This association remained consistent across different age and obesity subgroups. The study also identified a thoracolumbar kyphotic angle of 14° as the optimal cutoff point for defining poor functional outcomes. This angle was associated with high ODI and VAS scores, indicating that treatment should be initiated within the early stage following a VCF if the angle has reached this critical threshold.

## Figures and Tables

**Table 1:** Demographics (*n* = 161).

Item	Mean ± SD	Min.	Max.
Age	72.71 ± 5.61	65	91
BMI	26.48 ± 5.14	13.48	49.79
Spinal sagittal alignment			
CL	18.61 ± 14.23	−27	70
C7 slope	28.52 ± 11.1	−14.53	69.45
C2–7 SVA	17.21 ± 11.53	0	59.71
LL	38.99 ± 18.4	−8	88
LLL (L4-S1)	30.18 ± 13.15	−7	75
TL junction (T10-L2)	15.47 ± 10.68	1	60
SVA	64.75 ± 41.95	0	218.76
PI	55.13 ± 14.14	17.9	86.65
SS	32.76 ± 12.52	4.1	68.64
PT	22.37 ± 9.98	−9.53	46.93
PI-LL	18.48 ± 13.79	0.06	55.07
Functional outcome			
ODI	24.8 ± 9.38	10	48
VAS	5.25 ± 2.29	1	9

Note. BMI, body mass index; CL, cervical lordosis; LL, lumbar lordosis; LLL, lower lumber lordosis; TL, thoracolumbar; SVA, sagittal vertical axis; PI, pelvic incidence; SS, sacral slope; PT, pelvic tilt; ODI, Oswestry disability index; VAS, visual analogue scale; Min., minimum; Max., maximum. Data are presented as *n*, mean ± standard deviation, min. and max.

**Table 2:** Factors associated with ODI > 30 (*n* = 161).

Item	Crude		Adjusted	
	OR (95% CI)	*p* Value	OR (95% CI)	*p* Value
Age	0.96 (0.90, 1.02)	0.144	0.92 (0.84, 1.01)	0.086
BMI	1.04 (0.98, 1.11)	0.237	1.09 (0.98, 1.21)	0.107
CL	1.00 (0.98, 1.02)	0.877		
C7 slope	1.01 (0.98, 1.04)	0.566		
C2–7 SVA	0.99 (0.96, 1.02)	0.543		
LL	0.98 (0.97, 1.00)	0.068	0.96 (0.92, 1.01)	0.114
LLL (L4-S1)	1.01 (0.99, 1.04)	0.244		
TL junction (T10-L2)	1.27 (1.18, 1.37)	<0.001 *	1.28 (1.18, 1.39)	<0.001 *
SVA	0.99 (0.99, 1.01)	0.830		
PI	0.97 (0.94, 0.99)	0.008 *	0.98 (0.93, 1.03)	0.400
SS	0.97 (0.94, 0.99)	0.016 *	1.04 (0.96, 1.12)	0.327
PT	0.99 (0.96, 1.02)	0.442		
PI-LL	1.01 (0.99, 1.03)	0.495		

Note. BMI, body mass index; CL, cervical lordosis; LL, lumbar lordosis; LLL, lower lumber lordosis; TL, thoracolumbar; SVA, sagittal vertical axis; PI, pelvic incidence; SS, sacral slope; PT, pelvic tilt. Data are presented as odds ratio (95% CI). * *p* value ≤ 0.05 was considered statistically significant after test.

**Table 3:** Factors associated with VAS > 6 (*n* = 161).

Item	Crude		Adjusted	
	OR (95% CI)	*p* Value	OR (95% CI)	*p* Value
Age	0.95 (0.90, 1.01)	0.125	0.93 (0.86, 1.01)	0.070
BMI	1.04 (0.98, 1.11)	0.197	1.06 (0.98, 1.16)	0.146
CL	1.00 (0.98, 1.02)	0.998		
C7 slope	1.01 (0.98, 1.04)	0.465		
C2–7 SVA	0.99 (0.96, 1.02)	0.355		
LL	0.99 (0.98, 1.01)	0.447		
LLL (L4-S1)	1.02 (0.996, 1.05)	0.094	0.99 (0.95, 1.03)	0.672
TL junction (T10-L2)	1.16 (1.10, 1.22)	<0.001 *	1.17 (1.10, 1.23)	<0.001 *
SVA	1.00 (0.99, 1.00)	0.359		
PI	0.98 (0.96, 1.00)	0.111	1.00 (0.96, 1.04)	0.959
SS	0.98 (0.95, 1.01)	0.125	0.99 (0.94, 1.05)	0.926
PT	0.99 (0.96, 1.03)	0.734		
PI-LL	0.99 (0.98, 1.02)	0.948		

Note. BMI, body mass index; CL, cervical lordosis; LL, lumbar lordosis; LLL, lower lumber lordosis; TL, thoracolumbar; SVA, sagittal vertical axis; PI, pelvic incidence; SS, sacral slope; PT, pelvic tilt. Data are presented as odds ratio (95% CI). * *p* value ≤ 0.05 was considered statistically significant after test.

**Table 4:** Factors associated with ODI > 30 stratified by age and obesity (*n* = 161).

Item	Age ≤ 72 y/o (*n* = 89)		Age > 72 y/o (*n* = 72)		Nonobese (*n* = 93)		Obese (*n* = 68)	
	OR (95% CI)	*p* Value	OR (95% CI)	*p* Value	OR (95% CI)	*p* Value	OR (95% CI)	*p* Value
Age	-	-	-	-	0.88 (0.73, 1.05)	0.149	0.95 (0.82, 1.09)	0.453
BMI	1.15 (0.95, 1.38)	0.144	1.01 (0.83, 1.23)	0.947	-	-	-	-
CL	0.92 (0.83, 1.02)	0.116	0.97 (0.87, 1.08)	0.524	0.96 (0.88, 1.05)	0.386	0.91 (0.81, 1.03)	0.138
C7 slope	1.10 (0.97, 1.26)	0.143	0.93 (0.80, 1.09)	0.367	1.05 (0.94, 1.17)	0.434	0.99 (0.82, 1.19)	0.926
C2–7 SVA	0.91 (0.82, 1.01)	0.089	1.06 (0.94, 1.20)	0.356	0.93 (0.84, 1.03)	0.169	0.94 (0.82, 1.09)	0.419
LL	0.89 (0.80, 0.99)	0.043 *	1.11 (0.98, 1.25)	0.098	0.95 (0.87, 1.04)	0.300	1.01 (0.87, 1.18)	0.874
LLL (L4-S1)	0.96 (0.87, 1.07)	0.484	0.91 (0.80, 1.04)	0.167	0.94 (0.84, 1.05)	0.282	0.95 (0.85, 1.07)	0.422
TL junction (T10-L2)	1.53 (1.23, 1.90)	<0.001 *	1.35 (1.13, 1.61)	0.001 *	1.35 (1.16, 1.57)	<0.001 *	1.61 (1.23, 2.10)	0.001 *
SVA	1.00 (0.97, 1.04)	0.974	1.01 (0.97, 1.04)	0.731	0.99 (0.95, 1.03)	0.599	1.02 (0.98, 1.06)	0.249
SS	1.11 (0.94, 1.31)	0.223	0.98 (0.83, 1.15)	0.772	1.06 (0.91, 1.25)	0.453	0.97 (0.78, 1.20)	0.757
PT	0.97 (0.90, 1.06)	0.512	0.93 (0.84, 1.03)	0.158	0.92 (0.85, 0.99)	0.036 *	1.05 (0.94, 1.17)	0.373

Note. BMI, body mass index; CL, cervical lordosis; LL, lumbar lordosis; LLL, lower lumber lordosis; TL, thoracolumbar; SVA, sagittal vertical axis; SS, sacral slope; PT, pelvic tilt. Data are presented as odds ratio (95% CI). * *p* value ≤ 0.05 was considered statistically significant after test.

**Table 5:** Factors associated with VAS > 6 stratified by age and obesity (*n* = 161).

Item	Age ≤ 72 y/o (*n* = 89)		Age > 72 y/o (*n* = 72)		Nonobese (*n* = 93)		Obese (*n* = 68)	
	OR (95% CI)	*p* Value	OR (95% CI)	*p* Value	OR (95% CI)	*p* Value	OR (95% CI)	*p* Value
Age	-	-	-	-	0.88 (0.74, 1.05)	0.153	0.99 (0.88, 1.13)	0.914
BMI	0.99 (0.89, 1.11)	0.913	1.16 (0.96, 1.40)	0.120	-	-	-	-
CL	0.93 (0.87, 0.99)	0.047 *	0.95 (0.86, 1.05)	0.342	0.95 (0.86, 1.04)	0.268	0.92 (0.85, 1.002)	0.056
C7 slope	1.08 (0.99, 1.19)	0.086	0.99 (0.86, 1.15)	0.956	1.13 (0.98, 1.29)	0.086	0.98 (0.85, 1.12)	0.770
C2–7 SVA	0.94 (0.87, 1.01)	0.111	0.98 (0.87, 1.1)	0.692	0.85 (0.76, 0.96)	0.007 *	0.99 (0.89, 1.11)	0.853
LL	0.95 (0.87, 1.02)	0.169	1.05 (0.94, 1.17)	0.377	0.94 (0.87, 1.03)	0.182	0.98 (0.87, 1.11)	0.729
LLL (L4-S1)	1.00 (0.92, 1.09)	0.950	1.01 (0.92, 1.10)	0.897	0.96 (0.88, 1.06)	0.412	0.95 (0.86, 1.06)	0.376
TL junction (T10-L2)	1.24 (1.11, 1.38)	<0.001 *	1.18 (1.07, 1.30)	0.001 *	1.26 (1.12, 1.41)	<0.001 *	1.40 (1.17, 1.69)	<0.001 *
SVA	0.99 (0.97, 1.02)	0.630	1.01 (0.98, 1.04)	0.674	0.96 (0.92, 1.01)	0.095	1.00 (0.98, 1.04)	0.750
SS	1.04 (0.92, 1.18)	0.490	0.95 (0.81, 1.11)	0.524	1.08 (0.94, 1.24)	0.256	1.04 (0.87, 1.24)	0.638
PT	1.02 (0.96, 1.09)	0.517	0.95 (0.87, 1.04)	0.289	0.97 (0.9, 1.04)	0.363	1.03 (0.94, 1.12)	0.572

Note. BMI, body mass index; CL, cervical lordosis; LL, lumbar lordosis; LLL, lower lumber lordosis; TL, thoracolumbar; SVA, sagittal vertical axis; SS, sacral slope; PT, pelvic tilt. Data are presented as odds ratio (95% CI). * *p* value ≤ 0.05 was considered statistically significant after test.

## Data Availability

Data are contained within the article.
